# Evaluating a streamlined clinical tool and educational outreach intervention for health care workers in Malawi: the PALM PLUS case study

**DOI:** 10.1186/1472-698X-11-S2-S11

**Published:** 2011-11-08

**Authors:** Sumeet Sodhi, Hastings Banda, Damson Kathyola, Barry Burciul, Sandy Thompson, Martias Joshua, Eric Bateman, Lara Fairall, Alexandra Martiniuk, Ruth Cornick, Gill Faris, Beverley Draper, Martha Mondiwa, Egnat Katengeza, Lifah Sanudi, Merrick Zwarenstein, Michael J Schull

**Affiliations:** 1 Dignitas International, 2 Adelaide Street West, Suite 200, Toronto, M5H 1L6, Canada; 2 Department of Family and Community Medicine, Faculty of Medicine, University of Toronto, 263 McCaul Street, 5th Floor, Toronto, M5T 1W7; 3 Department of Family and Community Medicine, University Health Network, Toronto Western Hospital, 399 Bathurst Street, Toronto, M5T 2S8; 4 Research for Equity and Community Health (REACH) Trust, POB 1597, Lilongwe, Malawi; 5 Malawi, Ministry of Health Malawi, POB 3, Lilongwe, Malawi; 6 Zomba Central Hospital, Kamuzu Highway, Zomba, Malawi; 7 Knowledge Translation Unit, University of Cape Town Lung Institute, University of Cape Town, PO Box 34560, Groote Schuur 7937, South Africa; 8 Dalla Lana School of Public Health, University of Toronto, 155 College Street, Toronto, M5T 3M7, Canada; 9 George Institute for Global Health; University of Sydney, 341 George Street Sydney, 2000, Australia; 10 Sunnybrook Health Sciences Center; 2075 Bayview Ave, Toronto, M4N 3M5 Canada; 11 Nurses and Midwives Council of Malawi, POB 30361, Lilongwe, Malawi; 12 Department of Health Policy, Management and Evaluation, 155 College Street, Suite 425, Toronto, M5T 3M6, Canada; 13 IHCAR, Karolinska Institute, Nobelsveg 9, Stockholm, Sweden; 14 Department of Medicine, University of Toronto, 200 Elizabeth Street Toronto, M5G 2C4, Canada

## Abstract

**Background:**

Nearly 3 million people in resource-poor countries receive antiretrovirals for the treatment of HIV/AIDS, yet millions more require treatment. Key barriers to treatment scale up are shortages of trained health care workers, and challenges integrating HIV/AIDS care with primary care.

**The research:**

PALM PLUS (Practical Approach to Lung Health and HIV/AIDS in Malawi) is an intervention designed to simplify and integrate existing Malawian national guidelines into a single, simple, user-friendly guideline for mid-level health care workers. Training utilizes a peer-to-peer educational outreach approach. Research is being undertaken to evaluate this intervention to generate evidence that will guide future decision-making for consideration of roll out in Malawi. The research consists of a cluster randomized trial in 30 public health centres in Zomba District that measures the effect of the intervention on staff satisfaction and retention, quality of patient care, and costs through quantitative, qualitative and health economics methods.

**Results and outcomes:**

In the first phase of qualitative inquiry respondents from intervention sites demonstrated in-depth knowledge of PALM PLUS compared to those from control sites. Participants in intervention sites felt that the PALM PLUS tool empowered them to provide better health services to patients. Interim staff retention data shows that there were, on average, 3 to 4 staff departing from the control and intervention sites per month. Additional qualitative, quantitative and economic analyses are planned.

**The partnership:**

Dignitas International and the Knowledge Translation Unit at the University of Cape Town Lung Institute have led the adaptation and development of the PALM PLUS intervention, using experience gained through the implementation of the South African precursor, PALSA PLUS. The Malawian partners, REACH Trust and the Research Unit at the Ministry of Health, have led the qualitative and economic evaluations. Dignitas and Ministry of Health have facilitated interaction with implementers and policy-makers.

**Challenges and successes:**

This initiative is an example of South-South knowledge translation between South Africa and Malawi, mediated by a Canadian academic-NGO hybrid. Our success in developing and rolling out PALM PLUS in Malawi suggests that it is possible to adapt and implement this intervention for use in other resource-limited settings.

## Background

Although there have been significant gains in access to antiretroviral therapy (ART) and HIV care in the past decade, only one-third of the global population in need of ART currently have access to it (5.2 million out of 15 million) [1]. Substantial efforts are now underway to ramp up HIV/AIDS treatment in low and middle income countries, but there is a clear recognition that a major barrier to increased access is the lack of a sufficient, appropriately trained and mobilized healthcare workforce [2]. The workload inherent in a ramp-up of access to high quality HIV/AIDS services may also have unintended negative consequences on existing primary care services [3,4].

Since 2004, the Malawi Ministry of Health (MoH), Zomba District Health Office, and Dignitas International, a Canadian NGO, have collaborated to scale-up HIV/AIDS prevention and treatment in Zomba District, a primarily rural district of 670,000 people in south-eastern Malawi. Initially, an HIV/AIDS referral clinic was established in Zomba Central Hospital, followed by the development of a decentralised community-based model of HIV treatment, bringing treatment closer to the rural areas where most people requiring HIV/AIDS treatment live. More than 9000 adults and children now receive ART in the district, and HIV care has now been decentralized to about half of the district’s 30 public health centres, where ART provision is integrated with other outpatient primary care services. The burgeoning load of ART patients in rural health centres in Zomba District has placed an increasing burden on health care staff in those centres, where 50% of clinical posts are vacant. This reflects the situation across most of sub-Saharan Africa, which suffers from the lowest HCW-to-population ratio in the world [2]. This burden adds to the existing demand for non-HIV primary care, and the challenge of integrating HIV/AIDS services and other primary care services.

## The research

Dignitas International and REACH Trust, an independent Malawian health research organization, partnered with key stakeholders in Malawi and South Africa to develop and evaluate an intervention to support mid-level health care workers (HCW), such as nurses, medical assistants and clinical officers, in ART provision at the health centre level in Malawi. The intervention consists of a novel clinical tool and training approach known as “Practical Approach to Lung Health and HIV/AIDS in Malawi” (PALM PLUS). PALM PLUS is an expansion of earlier iterations, PALSA and PALSA PLUS (Practical Approach to Lung Health in South Africa), which themselves were originally adapted from the Health Systems Research Unit at the South African Medical Research Council, and the Knowledge Translation Unit at the University of Cape Town [5-7]. The PALSA and PALSA PLUS evaluation studies showed a significant increase in TB case detection, an increase in appropriate urgent referral among adult patients with cough or difficulty in breathing and an improvement in the provision of cotrimoxazole prophylaxis [8,9]. The successes and positive results demonstrated by the PALSA and PALSA PLUS teams were a primary motivating factor for the creation and mobilization of the PALM PLUS team.

The process of creating PALM PLUS involved adapting PALSA PLUS to the Malawian context through an iterative review process that included harmonization with existing Malawian treatment guidelines. This adaptation was done in full collaboration with the Ministry of Health, Malawian nurses and clinical officers, the Nurses and Midwives Council of Malawi, the Medical Council of Malawi, and other local clinical experts. Essentially, the PALM PLUS clinical tool is a paper-based algorithm that integrates and simplifies existing national guidelines for the management of HIV/AIDS, tuberculosis and other primary care conditions into a single, streamlined and user-friendly guideline, to support integration of primary care. Table 1 summarizes the sources for the content of the PALM PLUS clinical tool. Plans are in place for regular review of the content of PALM PLUS, and updating the clinical tool annually.

**Table 1:** Malawi National Guidelines Consulted in the Development of PALM PLUS.

Guideline Name	Edition/Date
Guidelines for the Use of Antiretroviral Therapy in Malawi	3^rd^ Edition, April 2008

Malawi Standard Treatment Guidelines	4^th^ Edition, 2009

National Tuberculosis Control Program Manual	6th Edition, 2007

Guideline for the Management of Malaria	August, 2007

Prevention of Mother to Child Transmission of HIV and Paediatric HIV Care Guidelines	2nd edition, July 2008

Management of HIV Related Diseases	2nd edition, April 2008

Guidelines for the Management of Sexual Assault and Rape in Malawi	November 2005

HIV/AIDS Counseling and Testing Guidelines For Malawi	2nd Edition, 2004

Management of Sexually Transmitted Infections Using Syndromic Management Approach	3rd Edition VI, March 2007

Malawi Essential Drug List	2009

The PALM PLUS clinical tool (a “job aid”) is accompanied by a training platform called educational outreach: facilitator led training that provides case-based, onsite, in-service education in the clinical settings where primary care providers work. This innovative strategy follows the proven, evidence-based model of PALSA PLUS [7]. Facilitators are also frontline health care workers, usually from the same health centre, who have received six or more intensive, interactive 1-2 hour health centre-based sessions. PALM PLUS training has been accredited by the MoH, and trainees receive credit for continuing professional development.

The research plan for PALM PLUS was developed with the intent to generate evidence that could be used to guide future decision-making by policy makers and program implementers in consideration of broader roll out of the intervention in Malawi. Thus, a pragmatic cluster randomized trial was conceived to compare usual care to the PALM PLUS intervention (clinical tool and educational outreach training approach together). Primary outcomes of the study are mid-level HCW job satisfaction and retention at health centres. Secondary outcomes are clinical outcomes at the health centre level that represent access to HIV/AIDS services, and integration of HIV/AIDS care with tuberculosis treatment and other primary care services provided at health centres.

**Figure 1:**
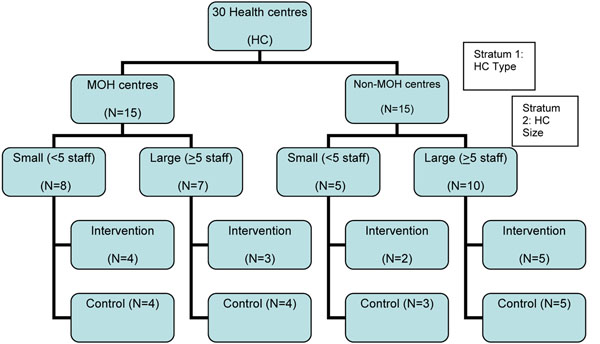
Randomization Flow Chart **HC type:** Non-MoH (Ministry of Health) facilities are either mission or institutional facilities. **HC size**: Small health centre have < 5 staff; large health centres have >5 staff

All public health centres in Zomba District (n=30) were randomized to either receive PALM PLUS or to serve as control sites (Figure 1). Health centres were first stratified by facility type, MoH or non-MoH, with non-MoH facilities being either mission (Christian Hospital Association of Malawi) or institutional facilities. Health centres were further stratified by size into small health centres with <5 clinical staff, and large health centres with ≥5 clinical staff.

In January 2010, 13 HCWs were trained as facilitators during a training-of-trainers workshop. Health centre-based educational outreach training sessions conducted by the facilitators at intervention sites started at the end of January and by June, all had completed the minimum required number of training sessions, a total of 132 altogether. Each HCW in the intervention sites received his or her own copy of the PALM PLUS guideline, and training to use the guideline as a desktop job aid. At many sites, staff continue to hold training sessions at their own initiative, well past the required minimum number of sessions.

Data collection started in January 2010 with the administration of a validated staff-satisfaction questionnaire to elicit levels of job and professional satisfaction, factors associated with planning to leave current employment, and levels of satisfaction of employment related amenities among HCW at control and intervention sites. A follow up survey was administered in May 2010, and another survey was conducted in February 2011. Collection of monthly health centre staffing data, intended to monitor HCW absentee rates, staffing levels and staff turnover, started in February 2011 and is ongoing. The first phase of qualitative data was collected in June 2010 through key informant interviews and focus group discussions to document training preferences and practices of HCWs at health centres. Health centre register data, health economics data and further qualitative data collection is underway, for ascertainment of additional primary and secondary outcomes. Results of this further data collection and analysis will be available in early 2012.

Ethics approval to conduct the study was obtained from the National Health Sciences Research Committee in Malawi. PALM PLUS training curriculum and materials were also reviewed and approved by the MoH. Since the staff satisfaction data were collected through a self-administered questionnaire, a consent form was attached to the questionnaire, and the HCWs were asked to read and sign the consent form before completing the questionnaire.

## Results and outcomes

In addition to generating evidence for policy makers and program implementers on the effectiveness of PALM PLUS, baseline and interim results and outcomes from this study will inform future versions of the PALM PLUS clinical tool and training curriculum. In the first phase of qualitative inquiry, health care workers were interviewed on their knowledge of PALM PLUS and availability of training opportunities. All participants reported that they had attended traditional trainings in the past 12 months, most of them as trainees and a few as trainers. Respondents from intervention sites described the PALM PLUS on-site training approach as participatory, while those who had participated in the workshop/seminar approach traditionally used by the MoH characterized themselves more as passive listeners during the training sessions. The PALM PLUS approach was thought to generate interest in the training content, as trainees and trainers interacted and shared patient-care experiences. PALM PLUS trainees recommended this approach to training because it accorded the participants an opportunity to plan their training schedule in a manner that avoided disrupting health service delivery at their facilities. The approach was also felt to relieve the participants of travel to distant training sites, which, according to some respondents, saved time and resources.

Participants in intervention sites felt the streamlined PALM PLUS tool empowered them to provide better health services to patients. They felt that the symptom-based approach used in PALM PLUS improved their recognition of the patient’s problems and their ability to offer appropriate treatment. Although respondents commended the tool as being useful in clinical practice, many indicated that its utilization had the chance of slowing down patient consultations, which was felt to be problematic at times when patient queues were long. Many respondents stated that once they became familiar with its content and algorithmic approach, they did not use the PALM PLUS clinical tool routinely at the point of care, as they gained familiarity with the material. They also stated that time pressures dissuaded them from routine use of the PALM PLUS clinical tool. This finding echoes qualitative evidence from South Africa, where experienced primary care providers indicated that the PALSA PLUS guideline was viewed more as a learning tool and reference guide, rather than a point-of-care “checklist”. However, nurses with less primary care experience used the PALSA PLUS guideline more regularly as a job-aid [10].

Almost all respondents mentioned allowances normally received during off-site MoH training as an important merit of that approach to training, and lack of the same in the PALM PLUS approach was considered to be a de-motivating factor for training. One trainer indicated that even though most training sessions were conducted during off-duty time, there was less participation from nurses than clinicians. This trainer felt that this might be due to of the lack of allowances provided to attend the PALM PLUS training sessions. When asked how they felt about attending an onsite training that might not give allowances, some HCW from control sites stated that they expected to receive allowances regardless of the site of training, onsite or off-site, as they felt this was a motivating factor for attending the training.

Baseline analysis of staff satisfaction surveys indicated no difference between HCW in intervention and control sites in terms of job satisfaction, professional satisfaction, likelihood of quitting in the next 12 months, plan to leave present job in the specified period, and perceived ease associated with finding a new job. The p-values for all variables were >0.05. Level of satisfaction was also assessed for different amenities among HCW: wages/salary, allowances, working hours, schedule flexibility, childcare availability, professional prestige, colleagues, staffing level, resources available, control over practice, pension, educational opportunities, responsiveness, clinical supervision, performance evaluation, and equipment/technology. There was no difference in terms of satisfaction with these amenities between HCW in intervention and control sites. There were high levels of overall satisfaction at intervention and control sites, however there were lower levels of baseline satisfaction on amenities in both groups.

Interim analysis of HCW absentee rates at health centres showed low absentee rates in all health centres, with attendance rates of greater than 95%. Of all the possible reasons for absences from their health centre post (sick leave, vacation, off-site training, other reason for being absent), being absent due to an off-site training was the most frequent reason cited by HCW (38% of all absent days).

Cumulative data on staffing levels for the first 12-month period of the study show that, on average, there were 8 clinical officers, 1 doctor, 19 medical assistants and 73 nurses per month in the control sites; and ten clinical officers, 16 medical assistants, 65 nurses and no doctor working per month in the intervention sites. Staff mobility monitoring shows that, on average, there were 3 staff and 4 staff departing from the control and intervention sites respectively, per month in the same 12-month period. On average, 2 new staff and 4 new staff joined control sites and intervention respectively, per month. The most common reason for departing a health centre was MoH initiated transfer, as indicated by 47 of 79 (59%) HCW departing from a health centre citing this reason. We hypothesize that staff turnover may have an impact on study outcomes, although we will attempt to adjust for this in future analyses.

## The partnership

The partnership is comprised of an international and inter-professional team including researchers, program implementers and policy makers from Canada, South Africa and Malawi. The Canadian partner, Dignitas International, is an academic-NGO hybrid which seeks to increase equitable access to effective HIV/AIDS-related prevention, treatment care and support in resource-limited settings through medical programs and health systems strengthening. In addition to its support to the MoH in Zomba District, Dignitas has developed a strong operational research capacity in Malawi, and has a research network centred at the University of Toronto. The South African partner, the Knowledge Translation Unit at the University of Cape Town Lung Institute, originally developed the PALSA (and subsequent iterations, such as PALSA PLUS) guidelines and rigorously tested their implementation; the intervention has now become national policy in South Africa.

The Malawian partners are REACH Trust and the Research Unit at the MoH. REACH Trust grew out of a research collaboration between the Malawian National TB Control Programme, the Department of Sociology of the University of Malawi, and the Liverpool School of Tropical Medicine, with experience in community-based experimental trials research. The Research Unit of the Malawi MoH has a long history of working with NGOs to carry out its mandate of evaluating MoH programming.

Additional Malawi-based team members include key national and district level Ministry of Health personnel, officials from the National TB Program, and representatives of Malawian health professional associations, including the Nurses and Midwives Council of Malawi.

Dignitas and the Knowledge Translation Unit have led the adaptation and development of the PALM PLUS guideline and training program, and protocol development for the trial. REACH Trust and the MoH Research Unit have led the qualitative and economic evaluations. Dignitas and the MoH have facilitated interactions among implementers, policy makers and decision-makers. This has led to a process of mutually reinforcing capacity building, where strengths and skills are shared among team members. Our strategies for successful collaboration include close and long-standing working relationships, shared resources, and frequent meetings, either in person or by internet teleconferencing.

## Challenges and successes

This initiative is an example of South-South knowledge translation between South Africa and Malawi, mediated by a Canadian academic-NGO hybrid. Our primary challenge has been that of combining program implementation in a “real world setting” with rigorous evaluation through a cluster randomized trial, which has raised issues of timing to synchronize implementation activities with evaluation activities, as well as issues of competing program priorities at health centres. This has been particularly challenging due to separate funding streams for implementation and research.

Although the project is still ongoing, a number of “lessons learned” have been noted.

1. In the pilot stage, health system innovations in resource-poor settings can benefit from the support of external (i.e. non-MoH) personnel; this can ease the burden on system managers, thereby making pilot projects viable. However, in planning and providing such support, attention must be paid to concerns of sustainability and local ownership, both of which are precursors to policy uptake. To this end, close consultation with key MoH stakeholders is essential.

2. In order to facilitate cooperation in matters such as filling in staff questionnaires, it is vital to keep health centre staff engaged and aware of the value of the study. Even before interim results are available, it is advisable to feed data back to the health centres on a regular basis in the form of simple descriptive statistics, e.g., graphs showing trends in patient load and disease burden.

3. Staff turnover, at all levels of the health system, represents a challenge to implementation and research. Consensus, once achieved, must be maintained through regular consultations and carefully tended handover processes as personnel change positions.

4. The integration of guidelines requires intensive collaboration, bringing together key personnel from multiple MoH Units and vertical disease programs to reconcile inconsistencies among various guidelines, to discuss real-world implementation challenges, and to explore ways of integrating primary care at the health centre level.

5. Using personnel who are experienced in PALSA/PALM-style guideline development and training carries enormous gains in project efficiency and effectiveness.

6. It is possible, helpful, and in some cases necessary to assemble a coalition of scientifically literate donor agencies in order to fund both the intervention and research activities associated with an innovative public health pilot program.

Health human resource shortages are widespread throughout sub-Saharan Africa. Our success so far with developing PALM PLUS in Malawi suggests that it is possible to adapt this tool for use in other resource-limited settings that are similarly affected with constraints in human resources for health. Future iterations of PALM PLUS will include regular revisions of guideline content as Malawian national treatment guidelines are updated. Further adaptations will target community health workers, to facilitate task-shifting for certain primary care conditions.

## List of abbreviations used

ANC: Antenatal Clinic; ART: antiretroviral therapy; CIDA: Canadian International Development Agency; DHO: District Health Office; HCW: health care workers; HIV/AIDS: Human Immunodeficiency Virus/Acquired Immunodeficiency Syndrome; MoH: Malawi Ministry of Health; NGO: non-governmental organization; PALM PLUS: Practical Approach to Lung Health and HIV/AIDS in Malawi; PALSA PLUS: Practical Approach to Lung Health and HIV/AIDs in South Africa; PMTCT: Prevention of Mother to Child Transmission; TB: Tuberculosis; UNAIDS: United Nations Programme on HIV/AIDS.

## Competing interests

The authors declare that they have no competing interests.

## Authors' contributions

MJS, SS, BB, ST, EB and MZ conceived the project. MJS, SS and BB led the drafting of the protocol and grant applications. RC, ST and LF led the guideline adaptation. GF and ST were responsible for adapting the training curriculum. HB, MJ, EB, LF, AM, and MZ participated in study design. DK, HB, MM and MJ helped design implementation, evaluation and content, and provided policy support. SS led the manuscript writing. BB assisted with manuscript writing. EK and LS assisted with data analysis and manuscript writing. All authors approved the final manuscript.
